# A Pipeline towards the Biochemical Characterization of the *Arabidopsis* GT14 Family

**DOI:** 10.3390/ijms22031360

**Published:** 2021-01-29

**Authors:** Lingling Xuan, Jie Zhang, Weitai Lu, Pawel Gluza, Berit Ebert, Toshihisa Kotake, Mengzhu Lu, Yuan Zhang, Mads H. Clausen, Kim L. Johnson, Monika S. Doblin, Joshua L. Heazlewood, Antony Bacic, Lili Song, Wei Zeng

**Affiliations:** 1 Sino-Australia Plant Cell Wall Research Centre, The State Key Laboratory of Subtropical Silviculture, College of Forestry and Biotechnology, Zhejiang A & F University, Lin’an 311300, China; linglingxuan@stu.zafu.edu.cn (L.X.); jiezhang@zafu.edu.cn (J.Z.); weitailu@stu.zafu.edu.cn (W.L.); lumz@zafu.edu.cn (M.L.); yz192814@ohio.edu (Y.Z.); K.Johnson@latrobe.edu.au (K.L.J.); M.Doblin@latrobe.edu.au (M.S.D.); T.Bacic@latrobe.edu.au (A.B.); 2 School of BioSciences, The University of Melbourne, Parkville, VIC 3010, Australia; pgluza@student.unimelb.edu.au (P.G.); berit.ebert@unimelb.edu.au (B.E.); jheazlewood@unimelb.edu.au (J.L.H.); 3 Division of Life Science, Saitama University, Saitama 338-8642, Japan; kotake@mail.saitama-u.ac.jp; 4 Center for Nanomedicine and Theranostics, Department of Chemistry, Technical University of Denmark, DK-2800 Kgs. Lyngby, Denmark; mhc@kemi.dtu.dk; 5 La Trobe Institute for Agriculture and Food, School of Life Sciences, Department of Animal, Plant, and Soil Sciences, AgriBio, La Trobe University, Bundoora, VIC 3086, Australia

**Keywords:** glycosyltransferase, *Arabidopsis*, plant cell wall, glycosylation, AGP, CAZy

## Abstract

Glycosyltransferases (GTs) catalyze the synthesis of glycosidic linkages and are essential in the biosynthesis of glycans, glycoconjugates (glycolipids and glycoproteins), and glycosides. Plant genomes generally encode many more GTs than animal genomes due to the synthesis of a cell wall and a wide variety of glycosylated secondary metabolites. The *Arabidopsis thaliana* genome is predicted to encode over 573 GTs that are currently classified into 42 diverse families. The biochemical functions of most of these GTs are still unknown. In this study, we updated the JBEI *Arabidopsis* GT clone collection by cloning an additional 105 GT cDNAs, 508 in total (89%), into Gateway-compatible vectors for downstream characterization. We further established a functional analysis pipeline using transient expression in tobacco (*Nicotiana benthamiana*) followed by enzymatic assays, fractionation of enzymatic products by reversed-phase HPLC (RP-HPLC) and characterization by mass spectrometry (MS). Using the GT14 family as an exemplar, we outline a strategy for identifying effective substrates of GT enzymes. By addition of UDP-GlcA as donor and the synthetic acceptors galactose-nitrobenzodiazole (Gal-NBD), β-1,6-galactotetraose (β-1,6-Gal_4_) and β-1,3-galactopentose (β-1,3-Gal_5_) to microsomes expressing individual GT14 enzymes, we verified the β-glucuronosyltransferase (GlcAT) activity of three members of this family (AtGlcAT14A, B, and E). In addition, a new family member (AT4G27480, 248) was shown to possess significantly higher activity than other GT14 enzymes. Our data indicate a likely role in arabinogalactan-protein (AGP) biosynthesis for these GT14 members. Together, the updated *Arabidopsis* GT clone collection and the biochemical analysis pipeline present an efficient means to identify and characterize novel GT catalytic activities.

## 1. Introduction

Glycosylation is one of the most important post-translational modifications (PTMs) of biological molecules. Most classes of organic macromolecules including polysaccharides, proteins, lipids, and nucleic acids as well as small molecules are composed of and/or can be modified with sugars [1]. Moreover, the cell surface of most organisms is coated with polysaccharides; the cell wall surrounding plant and fungal cells, the extracellular matrix of animal cells, bacterial cell envelopes, and the chitin exoskeleton of insects [2,3]. With few exceptions, glycosylation is an enzymatically catalyzed reaction that forms glycosidic bonds by transferring glycosyl residues from an activated donor, such as a nucleotide sugar or a lipid-linked intermediate, to a specific acceptor molecule [4]. This process requires glycosyltransferases (GTs) which can comprise 1–2% of the genome and consumes a significant proportion of cellular metabolism to maintain. 

Information relating to GTs has been deposited in the Carbohydrate-Active Enzyme (CAZy) Database (www.cazy.org). This database was created in 1998 and has since been expanded to include other sugar-related enzymes [5]. As of November 2020, the CAZy database has collected 768,132 genes from bacteria, viruses, archaea and 278 eukaryotic species into defined families and non-classified modules. However, only 2065 genes (less than 0.3%) have been functionally characterized. The model plant *Arabidopsis thaliana* is predicted to encode 573 GTs (42 GT families; approx. 2% of genome) compared to over 200 GTs encoded in the human genome (44 GT families; approx. 0.7% of total genes) [6,7]. The abundance of GTs in plants is attributed to a diversity of glycosylated secondary metabolites and a complex polysaccharide-rich cell wall. To facilitate functional approaches, a plant GT clone resource containing 403 CAZy-defined *Arabidopsis* GTs (http://gt.heazleome.org/) was developed [8], and these 573 *Arabidopsis* GTs are from the 42 defined CAZy families.

Small molecule glycosylation is mostly catalyzed by enzymes of the GT1 family [9]. Polysaccharides, such as cellulose the fibrillar component of the cell wall, are synthesized at the plasma membrane by cellulose synthase complexes (CSC) consisting of a “rosette” of cellulose synthase (CESA) catalytic sub-units belonging to the GT2 superfamily [10,11]. By contrast, most non-cellulosic polysaccharides are synthesized by GTs localized in the Golgi apparatus (GA) [12]. Xyloglucan, the dominant hemicellulose in dicot primary cell walls is synthesized by a suite of GTs including Cellulose synthase-like C glucan synthases (CSLCs, GT2) [13,14], xylosyltransferases (XTs, GT34) [15], galactosyltransferases (GalTs, GT47) [16], and fucosyltransferases (FUTs, GT37) [6,17,18,19]. Heteroxylan is the most dominant hemicellulose in the primary cell wall of the commelinid monocots (e.g., grasses) and the secondary cell walls of both monocots and dicots [20]. The xylan backbone synthesis process, although still not completely understood, involves members of the GT43 (IRX9), GT47 (IRX10, IRX14, IRX7/FRA8) and GT8 (IRX8/GAUT12, PARVUS/GATL1) families [21,22,23,24,25,26]. Glucuronosyltransferases (GUX, GT8) and arabinosyltransferases (AraT, GT61 in grasses) participate in xylan sidechain decoration [27,28]. Heteromannans, found in minor proportions in the primary vegetative walls of all land plants and as major components of the walls of some seed endosperms as well as the secondary walls of gymnosperms, are synthesized by CSLAs, a sub-class of the GT2 superfamily [29].

Acidic pectins are rich in galacturonic acid (GalA) and include homogalacturonan (HG), xylogalacturonan (XG), rhamnogalacturonan I (RG-I) and rhamnogalacturonan II (RG-II) [30]. Only a few GTs involved in pectin biosynthesis have been identified to date, including HG GalATs from the GT8 family [31,32], RG-I RhaTs (GT106) [33], RG-II XylT (GT77) and side chain galactan synthases (GT92) [34,35,36,37] and arabinan synthases (GT47) [38,39]. RG-II is considered one of the most complex polysaccharides in nature [40]. However, we only have rudimentary knowledge of the GTs responsible for RG-II biosynthesis. Functionally important glycoproteins such as arabino-galactan-proteins (AGPs) and extensins (EXTs) are also glycosylated in the endoplasmic reticulum/GA endomembrane system [41]. The GT31 family is responsible for the addition of the first galactose (Gal) to the hydroxyproline (Hyp) residues on AGPs and the synthesis of the β-1,3-galactan backbones of AGPs [38,42,43]. GT14 [44,45] and GT37 members [19] have been described that add two of the terminal sugars on AGPs, β-1,6-glucuronic acid (GlcA), and α-1,2-fucose (Fuc), respectively. The glycosylation of EXTs is catalyzed by members of other GT families: GT77 enzymes add α-1,3/1,5-arabinose (Ara) units [46]. The biochemically characterized *Arabidopsis* cell wall biosynthesis GTs are summarized in Supplementary Table S1.

The catalytic and acceptor specificities of most plant GTs are still unresolved and, therefore, an enzymatic characterization pipeline would greatly assist in this endeavor. The high-throughput characterization strategy for plant GTs could incorporate either genetic analyses or enzymatic assays or a combination of both. Reverse genetics of *Arabidopsis*, the study of the effect of gene disruption on plant phenotype, is a popular approach to determine gene function [47]. However, mutant phenotypes can often be either pleiotropic, redundant, or dependent on certain biotic or abiotic environmental conditions, thereby making the identification of the catalytic specificity of an enzyme challenging [48]. Hence, the biological and biochemical functions of the majority of these GTs is unresolved.

Heterologous protein expression combined with high-throughput enzyme assays can provide direct and robust evidence of a catalytic function. An effective GT enzymatic assay requires the right combination of nucleotide sugar donor, acceptor (derivatized sugar, oligosaccharide, glycolipid, or protein) and the active GT. Bioinformatics analysis of putative GTs is a valuable first step in determining which donors and acceptors to test in enzyme assays. Practical considerations such as availability then follow. Often, the exact acceptor is unknown and potentially complex and/or heterogeneous if derived from natural sources so a synthetic glycoconjugate is utilized instead. Choosing an effective heterologous protein expression system is vital to the success of the GT assay. It is generally recommended that a variety of heterologous expression systems, including prokaryotic (bacterial) and eukaryotic (e.g., yeast, animal or insect cells, or plants), are utilized to express, purify, and test the enzyme activities of plant GTs. The prokaryotic *Escherichia coli* expression system is a convenient and economical system and usually used to characterize GT1 members; however, due to the lack of post-translational modifications and/or appropriate folding, plant cell wall biosynthetic GTs expressed in *E. coli* are often inactive. Eukaryotic cells are most effective at expressing active plant GTs because they replicate both folding mechanisms and post-translational modifications. The yeast expression system *Pichia pastoris* has been utilized to confirm the catalytic activity of several plant GTs, including those involved in AGP [49], xylan [23], or heteromannan [50] biosynthesis (summarized in Supplementary Table S1). Animal cells can also be utilized to express plant GTs to obtain evidence of their functionality including *Arabidopsis* xyloglucan fucosyltransferase [51] and pectin GalAT (GAUT1) [31,32]. A plant expression system has significant technical advantages over non-plant systems. Previously, an active xylan synthase complex consisting of three proteins—IRX9, IRX14, (both GT43) and IRX10 (GT47)—was successfully expressed using *Agrobacterium*-mediated transformation of *Nicotiana benthamiana* [23]. The same plant expression system has also been widely used to identify the catalytic characteristics of GTs involved in the synthesis of other cell wall polysaccharides, such as (1,3;1,4)-β-glucan (mixed-linkage glucan) synthases [52,53,54], AGP hydroxyproline *O*-galactosyltransferases [43], and pectin RG-I rhamnosyltransferases [33].

Considering that a GT enzyme reaction requires the right combination of nucleotide sugar donor, appropriate acceptor and active GTs, identification of novel GT activity is challenging. Hence, a systematic platform to characterize the biochemical functions of plant GTs is needed to speed up functional assignment. In this study, we expanded the previous *Arabidopsis* GT clone collection [8] and characterized the catalytic activities of four GT14 family members to illustrate our approach. High-throughput subcellular localization of *Arabidopsis* GT14 members was highlighted in the previous GT collection study [8]. Some members of the GT14 family have previously been shown to exhibit glucuronosyltransferase (GlcAT) activity and therefore it was considered a good example to test our discovery pipeline [44,45]. In this study, all 11 members of the *Arabidopsis* GT14 family were transiently expressed in tobacco leaves and assayed using eight activated nucleotide sugar donors and three synthetic acceptor substrates mimicking AG chains on AGPs. Using this approach, an additional GT14 member was identified as a functionally active GlcAT using the acceptors galactose-nitrobenzodiazole (Gal-NBD) and galacto-oligosaccharides (β-1,6-galactotetraose (β-1,6-Gal_4_) and β-1,3-galactopentose (β-1,3-Gal_5_)). Our data provide further evidence that this enzyme family is involved in AGP biosynthesis and demonstrates that this strategy can be utilized to characterize GT function.

## 2. Results

### 2.1. Arabidopsis GT Collection

**Figure 1:**
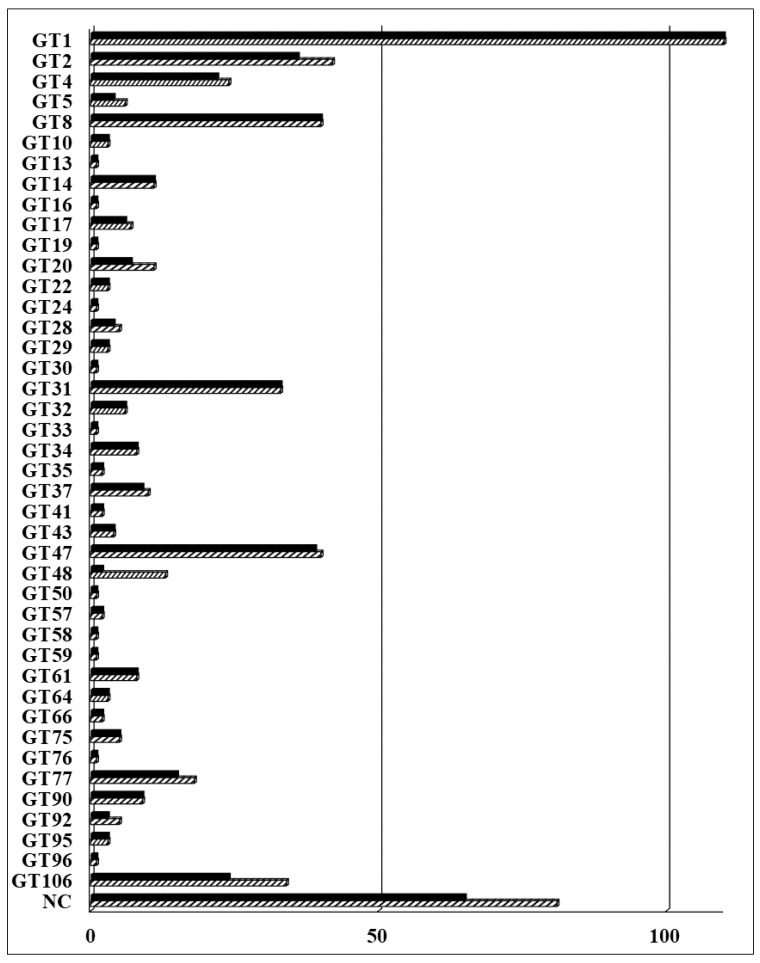
The collection of glycosyltransferases (GTs) from *Arabidopsis thaliana*. A total of 573 predicted GTs including 81 non-classified (NC) proteins are distributed in the 42 listed families; 508 genes have been cloned into pDONR223 vector for downstream analysis. The striped bars represent the total number of genes and the solid bars represent the number of cloned genes in each family. Detailed information of *Arabidopsis* GTs is listed in Supplementary Table S2.

In the previous collection, 403 *Arabidopsis* GTs were cloned into a Gateway-compatible binary vector for expression and functional analysis in plants [8]. We have updated the *Arabidopsis* GT collection in this study. A total of 565 *Arabidopsis* GTs is currently included in the CAZy database (www.cazy.org). Eight additional genes have been predicted to be putative GTs by the TAIR10 annotation (www.arabidopsis.org) or predicted to be non-classified GTs [55,56] and therefore have been added to our collection. A total of 573 predicted GTs and GT-like proteins is listed in Table S2. These include 492 GTs distributed across 42 families and 81 non-classified GTs (GTnc). In summary, 508 (89%) full-length GTs have been successfully cloned into the Gateway-compatible pDONR223 vector and are ready for downstream characterization (Figure 1). A total of 105 newly cloned GTs were added to the *Arabidopsis* collection reported by Lao et al. (2014) [8]. The coding sequence (CDS) of these genes are shown in the nucleotide (NT) sequence column of Table S2. The updated collection includes 65 GTnc (80%); proteins with a domain of unknown function (DUF) 266 (22), DUF 707 (11), DUF616 (6), DUF23 (4), and DUF288 (2). The remaining 20 GTnc members are listed without any specific classification. The 34 members of the DUF246 family are now re-classified as the GT106 family, with members seemingly being involved in pectin biosynthesis. Some have been identified as pectin RG-I rhamnosyltransferases [33] and others are involved with the biosynthesis of pectic arabinogalactans [57]. Additionally, four genes from the GT14-like family (DUF266) were cloned and compared with those in the previous collection [8].

### 2.2. Phylogenetic and Collinearity Analysis of Arabidopsis GT Families

**Figure 2:**
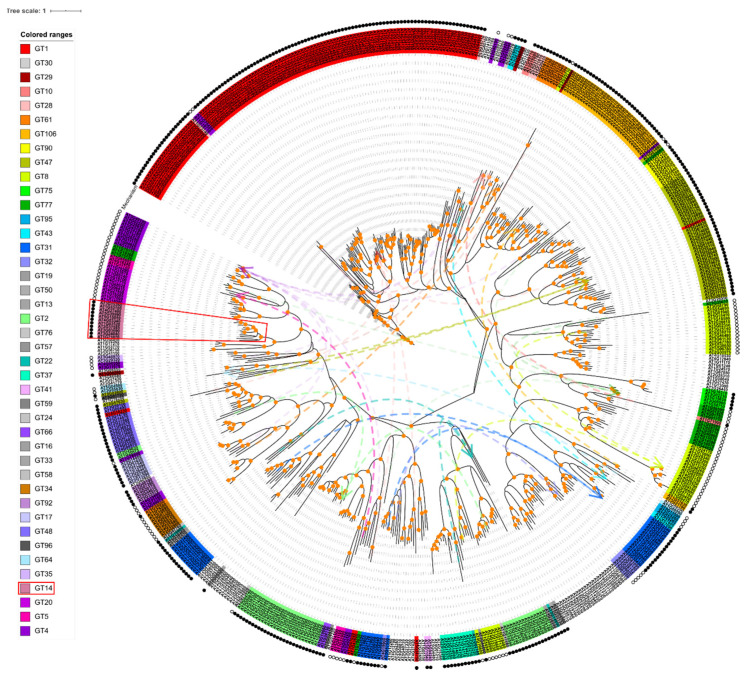
Phylogenetic analysis of the amino acid sequences of *Arabidopsis* GTs. Each GT family has a specific color, the genes without colored ranges are GTnc family, and GT14s are marked with red boxes. The legends with family-specific colors (family colors in all figures are hereby specified) are arranged in the order of family occurrence in the phylogenetic tree. Solid and hollow circles on the periphery represent inverting and retaining mechanism of each GT family, respectively. The branch points with orange circles indicate the bootstrap values over 60 and considered credible. Distant members of the same families on the tree are connected by dotted lines of family-specific colors.

To examine the phylogenetic relationships of *Arabidopsis* GT sequences, we constructed a maximum likelihood phylogenetic tree based on multiple sequences alignments of the full-length amino acid sequences of all 573 GTs from Supplementary Table S1 (see Figure 2). As expected, most GT family members clustered together forming either one or a few master blocks. However, some members showed scattered patterns in the following GT families: GT1, GT10, GT28, GT61, GT106, GT90, GT47, GT 75, GT77, GT95, GT32, GT2, GT37, GT41, GT66, GT34, GT92, GT17, GT48, GT35, GT14, and GT20. GT families 30, 19, 50, 13, 76, 59, 24, 16, 33, 58, and 96 have only one member. In addition, members in GT families 29, 8, 43, 31, 57, 22, 64, 5, and 4 are distributed into several different clades across the tree, indicating that diversified biochemical functions of their members are more likely. There are more inverting GTs (30 families, 369 genes) than retaining GTs (12 families, 123 genes) as shown in Figure 2. Since the biochemical functions of GTnc are not characterized, the inverting/retaining mechanisms of GTnc are not assigned.

**Figure 3:**
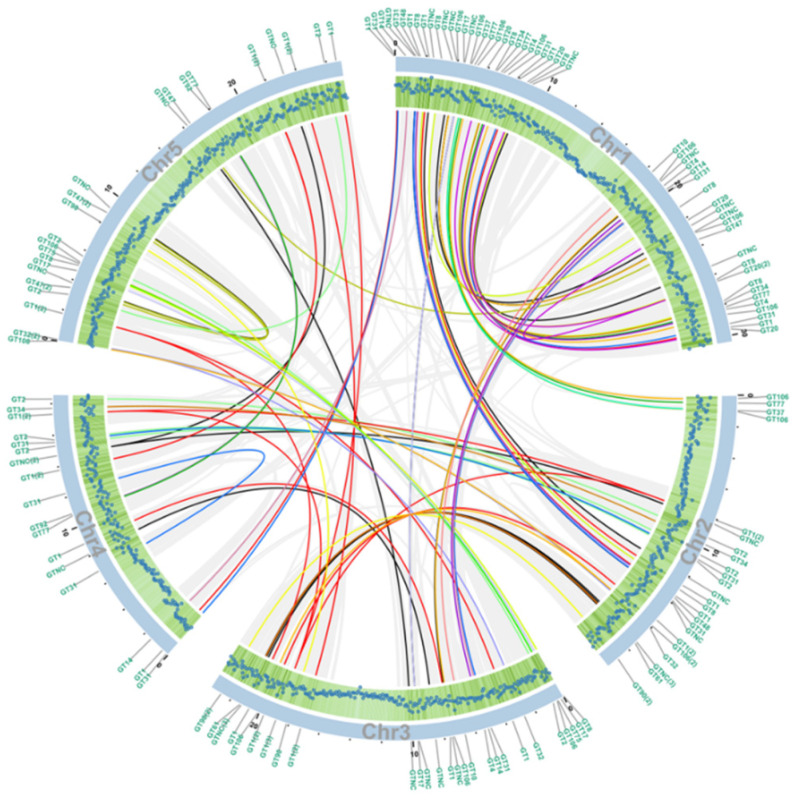
Collinearity analysis of the GT genes between pairs of *Arabidopsis* chromosomes. The scales on the outside of chromosome circle segments in blue represent the length of the chromosome. Each blue dot in the green shaded area shows the gene density at 100 kb intervals, the further towards the outside the greater the density. Collinear blocks of non-GT genes are linked by grey lines, GT family genes are linked by lines of family-specific colors indicated in Figure 2. The GT family to which the gene belongs is marked on the perimeter.

If the order of a series of at least five genes in one block of a chromosome is identical to that of homologous genes in another part of the genome, the homologous genes are considered to share collinearity [58]. Hence, these genes are more likely to have similar functions compared to genes that are not collinear. Out of 52,270 predicted *Arabidopsis* proteins according to the TAIR10 database, 6409 proteins have been shown to share collinearity, accounting for 12.26% of all proteins, shown by light grey lines in Figure 3. Among all the collinearity blocks, 84 pairs belong to the GT superfamily (Table S3), indicating the potential functional similarity of these enzymes. The gene density on each chromosome correlates with the number of GTs (Figure 3). The GT distribution of each chromosome is shown in Figure S1.

### 2.3. In Silico Analysis of the Arabidopsis GT14 Family

**Figure 4:**
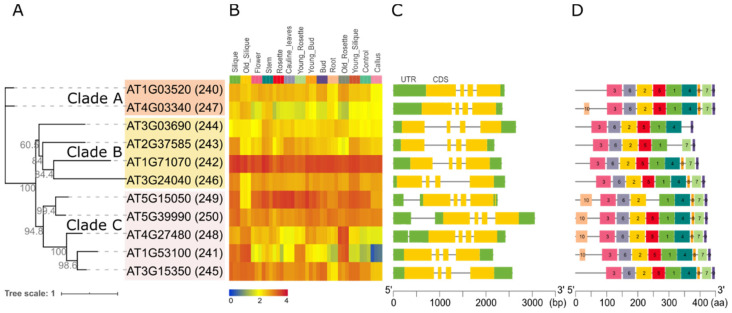
Bioinformatic analyses of the 11 *Arabidopsis* GT14 family genes and their translated proteins. (**A**) ML phylogenetic tree with bootstrap values of 1000 (SH-aLRT test). The sequences were divided into three distinct clades (A, B, and C) shaded in orange, yellow and pink, respectively. (**B**) AtGT14 family genes expression in different tissues during the growth and development processes of *A. thaliana*. The expression data were taken from GSE34188 [59] in the NCBI Gene Expression Omnibus (GEO) database (https://www.ncbi.nlm.nih.gov/geo/). (**C**) Gene structure analysis. The green blocks represent UTR, the yellow blocks represent coding sequence (CDS), the grey lines represent introns. (**D**) Protein sequence motif analysis using MEME with setting the maximum number of motifs = 10. The motifs mean the approximate sequence pattern in a group of homologous genes.

A maximum likelihood phylogenetic tree of the *Arabidopsis* GT14 family was constructed to obtain further insight into their relatedness and potential function (Figure 4A). The 11 genes (named 240–250) are distributed into 3 clades: AT1G3520 (240) and AT4G03340 (247) form clade A (orange); AT3G03690 (244), AT2G37585 (243), AT1G71070 (242), AT3G24040 (246, AtGlcAT14D) form clade B (yellow); AT5G15050 (249, AtGlcAT14B), AT5G39990 (250, AtGlcAT14A), AT4G27480 (248), AT1G53100 (241), and AT3G15350 (245, AtGlcAT14E) form clade C (pink).

The expression pattern of each GT14 gene in different tissues are shown in a heat map in Figure 4B. The genes AT1G71070 and AT5G15050 have high expression across all tissues and AT4G03340 has the lowest expression across most tissues. AT1G53100 shows highest expressions in senescent organs such as old silique and rosette. The gene structure (intron, exons, 5′ and 3′ UTRs) and protein motif distribution of GT14 members are shown in Figure 4C,D, respectively. Notably, all of the GT14 family genes have a similar exon-intron pattern with three introns and four exons with clade members showing more similarity to each other than with other clade sequences (Figure 4C). Similarly, the GT14 proteins share most of the 10 predicted protein motifs identified with MEME. Motif 4 is missing in AT2G37585, motif 5 missing in AT5G15050, motif 7 is missing in AT3G03690, motif 8 is missing in AT2G37585 and AT3G03690. Interestingly, motif 10 exists in only one protein (AT4G03340) of clade A and all proteins of clade C except AT3G15350.

### 2.4. Heterologous Expression of Arabidopsis GT14 Family Proteins in N. benthamiana

For function assays, C-terminal YFP-tagged versions of the 11 AtGT14 family members were individually expressed in a transient manner in *N. benthamiana* leaves and microsomal membranes (MMs) prepared 3 days post-inoculation. A GFP construct was used as negative control. The level of expression of each fusion protein was detected with anti-GFP antibody using western blotting (Figure S2). Each of the tagged GT14 proteins was expressed and was of the expected size of around 75 kDa (between 44–52 kDa + YFP (approx. 27 kDa), Figure S2). The GFP negative control showed a band of the expected molecular weight of approx. 27 kDa. This confirmed the successful expression of all 11 AtGT14 family proteins in tobacco leaves.

### 2.5. Identification of Arabidopsis GT14 Family Members as Glucuronosyltransferases (GlcATs)

**Figure 5:**
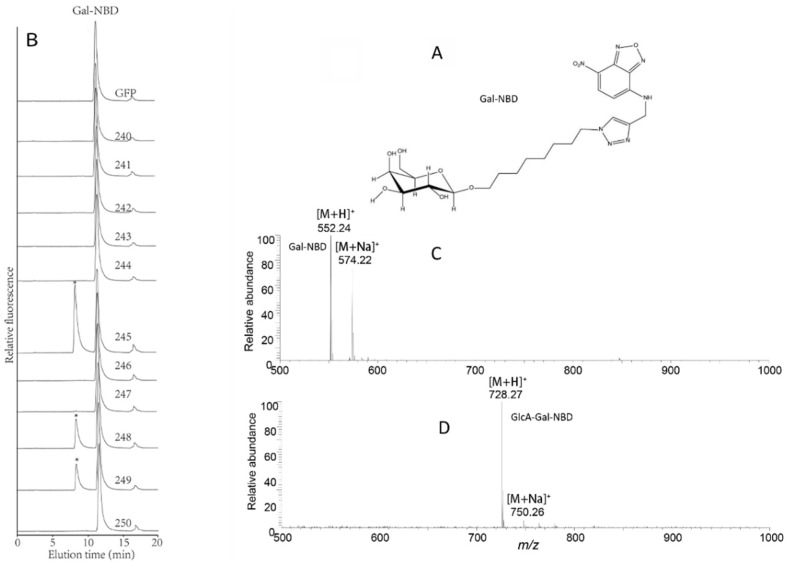
Catalytic activity of 11 *Arabidopsis* GT14 family members using UDP-GlcA as the donor and β-Gal-NBD as the acceptor. (**A**) The molecular structure of the acceptor β-Gal-NBD. (**B**) The microsomal membranes of the negative control (GFP) and the 11 recombinant AtGT14 family genes (240–250) expressed in *Nicotiana benthamiana* as enzymes were incubated with UDP-GlcA donor and β-Galactose-nitrobenzodiazole (β-Gal-NBD) acceptor for 2 h to test glucuronosyltransferase (GlcAT) activity. The reaction products were analyzed by RP-HPLC with fluorescence detection. The product peaks were labeled with an asterisk (*). The 11 recombinant AtGT14 family genes numbered as 240–250 (240: AT1G03520, 241: AT1G53100, 242: AT1G71070, 243: AT2G37585 (AtGlcAT14C), 244: AT3G03690, 245: AT3G15350 (AtGlcAT14E), 246: AT3G24040 (AtGlcAT14D), 247: AT4G03340, 248: AT4G27480, 249: AT5G15050 (AtGlcAT14B), 250: AT5G39990 (AtGlcAT14A)). The fluorescent tag NBD is detected by a fluorescence detector with λex = 470 nm, λem = 540 nm. (**C**) ESI-MS spectrum of the acceptor Gal-NBD. (**D**) The LC-ESI-MS spectrum of the reaction products catalyzed by the recombination AT3G15350 (245) microsomes.

Previous studies have shown that AtGlcAT14A (AT5G39990, 250), AtGlcAT14B (AT5G15050, 249), and ATGlcAT14C (AT2G37585, 243) have GlcAT activity and are involved in the biosynthesis of type II arabinogalactan (AG), a glycan decoration found on AGPs [44,45]. Recently, AtGlcAT14D (AT3G24040, 246) and AtGlcAT14E (AT3G15350, 245) were also shown to be essential in the glucuronidation of AGs [60]. Here, we sought to investigate the putative GlcAT activity of other members of the AtGT14 family using a suite of synthetic acceptors designed to mimic endogenous AGP acceptors. We utilized MMs prepared from *N. benthamiana* leaves expressing each of the 11 AtGT14 enzymes or the negative control (GFP) in enzymatic assays. Initially, UDP-GlcA and β-galactose-nitrobenzoxadiazole (β-Gal-NBD) were used as donor substrate and acceptor, respectively. β-Gal-NBD has been demonstrated to act as a synthetic mimic of Gal-containing oligosaccharides observed in arabino-3,6-galactan (type II AG) sidechains of AGPs [38]. The chemical structure of β-Gal-NBD is shown in Figure 5A and includes an eight-carbon spacer between β-Gal and NBD to minimize any negative impacts of the tag on enzyme activity, a commonly applied strategy to address steric hinderance issues [61]. Using RP-HPLC to separate the products of the assay, β-Gal-NBD was found to elute at 11.5 min in all samples. A significant peak was also observed to elute at 8.1 min amongst the assay products of three GT14 member samples (AT3G15350 (245), AT4G27480 (248), and AT5G15050 (249)), with AT3G15350 (245) showing the highest activity. These results demonstrate that Gal-NBD is able to be utilized as acceptor in the GlcAT assay (Figure 5B). The fraction between 7 and 10 min, including the 8.1 min peak of the enzymatic reaction of AT3G15350 (245), was collected and further analyzed by ESI-MS. LC-MS analysis revealed the presence of glucuronosylation of β-Gal-NBD from protonated acceptor (Gal-NBD hydrogen adduct with m/z 552.24 and sodium adduct with m/z 574.22, Figure 5C) with the hydrogen adduct being the most abundant pseudomolecular ion (GlcA-Gal-NBD hydrogen adduct with m/z 728.27 and sodium adduct [M + Na]^+^ with m/z 750.26) (Figure 5D). This result confirms that AtGlcAT14B and AtGlcAT14E have GlcAT activity and a new GT14 member AT4G27480 (248) was also found to share this activity.

**Figure 6:**
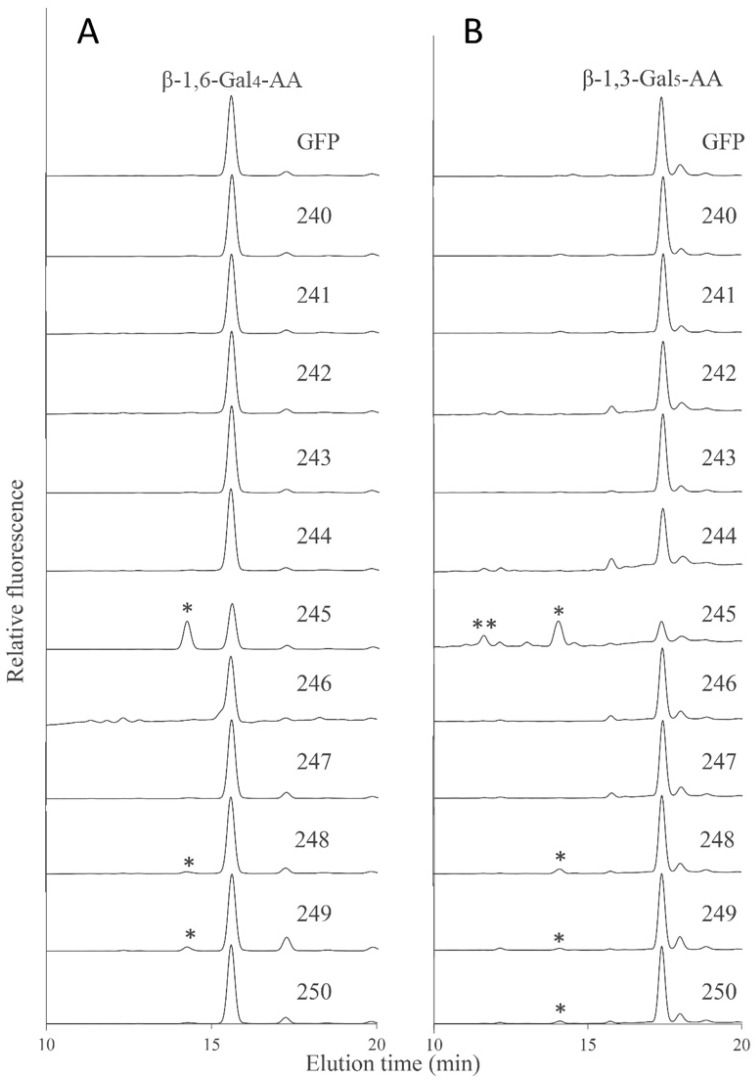
Glucuronosyltransferase (GlcAT) activity of the 11 recombinant AtGT14 family genes (240–250) using β-1,6-Gal_4_-AA (**A**) and β-1,3-Gal_5_-AA (**B**) as acceptors. The reaction products were analyzed by RP-HPLC using fluorescence detection (λex = 320 nm; λem = 420 nm) and different products eluting at 11.6 min and 14.1 min labeled with asterisks (* and **), respectively. GFP is the negative control. These results indicate that the gene 245 (AT3G15350) catalyzes an addition of GlcA residues to both β-1,3- and β-1,6-linked galactans.

In order to further characterize the biochemical activity of GT14 enzymes, the galacto-oligosaccharides β-1,6-Gal_4_ and β-1,3-Gal_5_ were fluorescently tagged with anthranilic acid (AA) and then used as acceptors in similar enzymatic assays using the GT14-containing MMs as enzyme source. RP-HPLC chromatograms showed that the AT3G15350 (245), AT4G27480 (248), and AT5G15050 (249) MMs had additional peaks compared to the negative control suggesting that the β-1,6-Gal_4_-AA acceptor was able to be utilized by these AtGT14 enzymes, with MM extracts containing AT3G15350 (245) showing significantly higher GlcAT activity than AT4G27480 (248) and AT5G15050 (249) (Figure 6A), similar to the pattern observed with the Gal-NBD acceptor (Figure 5B). The products of enzymatic assays in which β-1,3-Gal_5_-AA was used as acceptor were analyzed in the same way, with AT3G15350 (245), AT4G27480 (248), AT5G15050 (249), and AT5G39990 (250) MM extracts shown to have GlcAT activity with the detectable glucuronosylated products eluting at 11.6 min and 14.1 min (Figure 6B). Thus, *Arabidopsis* GT14 family members including AT5G39990 (AtGlcAT14A), AT5G15050 (AtGlcAT14B), AT3G15350 (AtGlcAT14E), and AT4G27480 show the GlcAT activity using the three different acceptors, thereby verifying the previously reported activities of AtGlcAT14A, B and E and demonstrating that the family-wide approach led to the identification of another GT14 family member (AT4G27480) having similar GlcAT activity.

### 2.6. Characterization of the Donor Substrate Specificity of the AtGT14 Recombinant Proteins

**Figure 7:**
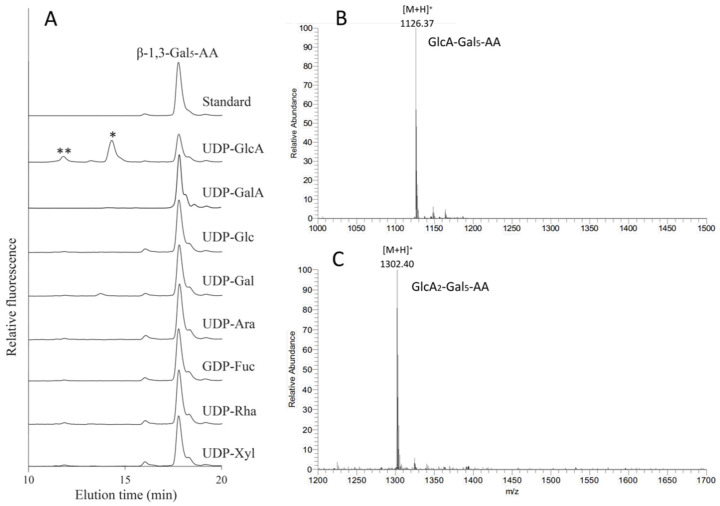
Donor specificity of AT3G15350 (245). (**A**) Eight NDP-sugars were tested for GT activity using *N. benthamiana* microsomes expressing AT3G15350 in the presence of the β-1,3-Gal_5_-AA acceptor. The reaction products were analyzed by RP-HPLC using fluorescence detection (λex = 320 nm; λem = 420 nm) and are labeled with asterisks (* and **). The fraction between 13 and 15 min (*) (**B**) and the other fraction between 10.6 and 12.6 min (**). (**C**) collected for analysis by LC ESI-MS. From the molecular ion species we concluded that the peaks at 14.1 min and 11.6 min with the m/z values of 1126.37 (**B**) and 1302.40 (**C**) were the predicted pseudo-molecular ions [M + H]^+^ of GlcA-Gal_5_-AA and GlcA_2_-Gal_5_-AA, respectively.

Among the 11 *Arabidopsis* GT14s, AT3G15350 (245, ATGlcAT14E) was demonstrated to have the highest GlcAT activity using Gal-containing acceptors and was therefore used in nucleotide sugar donor specificity assays. Seven additional nucleotide sugar donors (UDP-GalA, UDP-Glc, UDP-Gal, UDP-Ara*p*, GDP-Fuc, UDP-Rha, and UDP-Xyl) were tested in the presence of the β-1,3-Gal_5_-AA acceptor. However, only UDP-GlcA was an active substrate donor, while other donors did not show any glycosylation activity (Figure 7A). The fractions from 10.6 to 12.6 min and 13 to 15 min were collected for analysis by LC ESI-MS. The peaks at 14.1 min and 11.6 min with the m/z values of 1126.37 (Figure 7B) and 1302.40 (Figure 7C) were the predicted pseudomolecular ions [M + H]^+^ of GlcA-Gal_5_-AA and GlcA_2_-Gal_5_-AA, respectively. This result indicates that up to two GlcA residues were incorporated by AtGlcAT14E onto the β-1,3-Gal_5_-AA acceptor whereas addition of only one GlcA residue was incorporated using the β-1,6-Gal_4_-AA acceptor.

## 3. Discussion

### 3.1. Plant GT Families Are Highly Diverse

Despite significant progress being made in the area of glycobiology, many questions still remain unresolved, especially in the plant field. Among the GTs classified into families, only 151 GTs have been annotated as characterized in *Arabidopsis* according to the CAZy database. In the human genome, only one GT is assigned as non-classified in comparison to 81 in *Arabidopsis*, hence indicating that glycosylation is a far more complex process in plants and further work is required to determine the biochemical activities of these GTs. 

Among the *Arabidopsis* non-classified GTs, the DUF266 proteins are plant-specific. These proteins are distantly related to the GT14 family [62] and therefore they were named as the GT14-like family [63]. Both DUF266 and GT14 enzymes share the similarity of a core 2 β-1,6-N-acetylglucosaminyltransferase C2/4GnT (C2GnT-2) from animal GTs, indicating that the GT14 and DUF266 families may have evolved from the same ancestral gene/s [63,64]. A few studies have used genetic tools to uncover the biological functions of DUF266 proteins. For example, the rice DUF266-containing protein BC10 regulates cell wall biosynthesis as the mutant shows a brittle stem phenotype [65] and another rice DUF266 protein RSE1 impacts leaf senescence [66]. Future analysis and biochemical characterization of members of the DUF266 family will provide further information on the functions of these proteins.

### 3.2. Evolution of GTs Correlated with Their Enzymatic Functions

The phylogenetic relationships of each *Arabidopsis* GT are reflected in Table S3 and Figure 2. While most GT families are clustered together forming one or more master blocks in the phylogenetic tree, there are few exceptions where several isolated genes from the same family showed a scattered pattern (GT1, 10, 28, 61, 106, 90, 47, 75, 77, 95, 32, 2, 37, 41, 66, 34, 92, 17, 48, 35, 14, 20) (Figure 2) [56,62,67,68]. 

Among these GT families, the GT1 family is the largest and the most ancient and its members are known to generally glycosylate small hydrophobic molecules. For example, UGT72Es are capable of conjugating lignin monomers and related metabolites (such as aldehydes, coniferyl, and sinapyl alcohols) [69]; UGT76C1, UGT76C2 and UGT85A1 are cytokinin-specific glycosyltransferases [70]; UGT78K1 and UGT78D2 are flavonoid 3-O-glucosyltransferases [71]; UGT80A2 and UGT80B1 are enzymes that catalyze the synthesis of steryl glycosides [72]. It should be noted that two GT28 members (AT1G73740 and AT4G16740) and two GT4 members (AT1G75420 and AT1G19710) are clustered with GT1 (Figure 3, Table S3) [73]. There are three genes in the GT10 family. AT1G49710 and AT3G19280 form a collinearity gene block encoding enzymes with the same biochemical function of α-1,3-l-FucTs, whereas the third gene (AT1G71990) encodes α-1,4-l-FucT (Figure 3, Table S3) [74,75], indicating the gene pairs sharing collinearity may have similar biological functions. Members in the GT106 family are annotated as O-FucTs and RG-I RhaTs. The functions of GT47 genes are annotated as exostosin (α-N-acetylglucosaminyltransferase) family proteins, except for AT5G03800 (Embryo Defective) [75] and AT3G57630 (Extensin Arabinose Deficient Transferase) [76]. The GT77 family can be divided into two main clades, Reduced Residual Arabinoses (RRAs) and Rhamnogalacturonan Xylosyltransferases (RGXT1), except for AT2G02060 (Homeodomain-Like Superfamily Protein) and AT2G35610 (Extensin Arabinosyltransferase) [76]. Noticeably, two ‘orphan’ GTs—one from the GT77 family AT1G70630 (Reduced Arabinose Yariv1, RAY1) [46] and the GT32 protein (AT4G19900)—are in clades separated from the rest of the family. The GT2 family is divided into two main clades with the exception of AT1G20575 [77] and AT2G39630 (Dolichol Phosphate Synthases), indicating the distinct origin of these GTs. The GT14 family will be discussed in detail below which is shown in Figure 2 (prominently in pink and marked with a red box) and in Figure 4A with three clades (A, B, and C).

For GT29, 8, 43, 31, 57, 22, 64, 5 and 4, the differences in the function of their members are predicted from protein annotation [56,62,68,78] and in part experimentally verified (Figure 2). The three members (AT1G08280 (AtGALT29A), AT1G08660 (MGD2), AT3G48820) of the GT29 family are separated on the phylogenetic tree, suggesting they have functionally diversified. Indeed, the annotation of three enzymes in the GT29 family are different according to the CAZy database although their exact biochemical functions are to be investigated. The GT8 family has 40 members with various glycosylation activities including xylan biosynthesis, sphingolipid glucuronosylation, and HG synthesis. The GT43 family is divided into two clades, including IRX 9 (AT1G27600, AT2G37090) and 14 (AT4G36890, AT5G67230) [79] both involved in xylan backbone biosynthesis with distinct roles [23]. The GT31 family has been characterized as GalTs and falls into three clades, consistent with the phylogenetic analysis [41].

### 3.3. The Expression System and Enzyme Assay for GT Biochemical Characterization

There are numerous expression systems that have been utilized to characterize GTs, including bacteria, the yeast *Pichia*, and the human cell line HEK 293 [80,81,82]. There is not one ideal expression system to characterize the biochemical functions of all GTs. However, many non-plant expression systems result in a lack of detectable enzymatic activity that may either be due to inappropriate post-translational modification and/or the absence of appropriate endogenous co-factors. In this study, we took advantage of the *N. benthamiana* (tobacco) transient expression system, which is one of the most efficient systems for expressing plant proteins [83]. YFP-tagged versions of the 11 AtGT14 family members were all able to be expressed at detectable levels in tobacco leaves using *Agrobacterium*-mediated transformation (Figure S2). Other GTs could also be expressed in the same system and verified with western blot to accumulate to detectable levels for the downstream of GT biochemical assays. The MMs prepared from infiltrated leaves were used directly in enzyme assays as crude enzyme preparations but if necessary, can be further purified using GFP-trap beads for activity assays and other purposes, such as identifying interacting proteins. The tobacco expression system can easily accommodate co-infiltration of multiple *Agrobacterium* vectors without the need to construct a complex multigene expression vector [23]. Therefore, the tobacco expression system can be used as a convenient and efficient method to screen the enzymatic activity of plant GTs. 

The success of the assay in defining GT specificity depends on the precise combination of an appropriate nucleotide sugar donor, a specific molecule such as an oligosaccharide as an acceptor and an active GT preparation as an enzyme source. A particular bottleneck for the characterization of GTs lies in obtaining the right acceptors. Plant-derived carbohydrate oligosaccharides are usually complex and difficult to use directly in enzyme assays. For example, β-Galactosyl Yariv specifically recognizes AGPs and can be used in their purification [84]. However, as highly heterologous and complex macromolecules, AGPs are difficult to be directly used as acceptors for enzyme assays. Chemical synthesis provides a solution to this problem, although this approach is not straightforward. In future, the chemical synthesis of oligosaccharide fragments mimicking polysaccharides will be of great benefit for the identification of plant cell wall GTs [6].

In this study, we chose the *Arabidopsis* GT14 family to test the pipeline as multiple Gal-containing acceptors were available, were utilized in previous GT14 enzyme assays, and were shown to act as acceptors for multiple GT14 enzymes [44].

### 3.4. Members of the AtGT14 Family Display GlcAT Activity

AGPs are proteoglycans within the hydroxyproline-rich glycoprotein (HRGP) superfamily with type II arabino-3,6-galactans comprising up to 90% (*w*/*w*) of the molecule [85]. As a major part of AGPs, the type II AG moieties play a pivotal role in various plant growth and developmental processes and the biosynthesis of these complex structures requires multiple GTs [86]. AG sugar chains have a backbone of β-1,3-galactan, with the sidechains of β-1,6-galactan that have Ara, Rha, Fuc, and GlcA as branch terminal residues. Additionally, some GlcA residues are attached directly to the β-1,3-galactan backbone [86]. The GT31 family has been shown to be associated with the synthesis of the AGP galactan backbone and the synthesis of sidechains has been attributed to members of the GT14 (GlcAT), GT29 (β-(1,6)-GalT), GT37 (FucT), and GT77 families (AraT) [87]. Here, we provide evidence that at least one new member, AT4G27480 from the GT14 family, is a functional GlcAT and can add GlcA to both β-1,3- and β-1,6-galacto-oligosaccharides (Figure 6A,B, respectively) in addition to the Gal-NBD acceptor (Figure 5B). AT5G39990 (AtGlcAT14A) only incorporates GlcA onto β-1,3- but not β-1,6-galacto-oligosaccharides. Longer β-1,6-galacto-oligosaccharides could also be tested in the future if available. Notably, the MM extracts from plants expressing AtGlcAT14E had a greater activity than those from plants expressing either AtGlcAT14A, AtGlcAT14B, or AtGlcAT14C enzymes previously identified to possess GlcAT activity (Figure 6) [44,45]. This result suggested that AtGlcAT14E is a GlcAT, catalyzing the addition of GlcA residues onto β-1,3- and β-1,6-linked galactans of AGPs. MM extracts from plants expressing AT4G27480 also showed some GlcAT activity with the same acceptors (Figure 6A,B), indicating it too is likely a GlcAT. Thus, our data expand the number of AtGT14 family members with demonstrated GlcAT activity to six.

In a previous study, AtGlcAT14C was shown to function as a GlcAT [45], a finding that is inconsistent with our results (Figure 6A). However, in vitro enzyme activity may vary between laboratories, expression systems, and enzyme assays including the availability of acceptors. Four proteins of clade C (including previously characterized AtGlcAT14A, B and E) demonstrated GlcAT activity using galactosyl-oligosaccharides as acceptors. Neither clade A nor clade B (including AtGlcAT14C and AtGlcAT14D) show any GlcAT activity, indicating enzymes from GT14 clade A and B may be involved in other glycosyltransferase activities. We have also expressed nine poplar GT14 genes and their enzymatic activity is consistent with the current *Arabidopsis* study (data not shown). It is somewhat surprising that no GlcAT activity was detected for AT1G53100 given the similarity of the sequences within clade C of the GT14 family (Figure 4A). A possible explanation may be that the level of this recombinant GT14 enzyme was insufficient to generate a detectable amount of reaction product or it needs additional co-factors such as other AGP biosynthesis protein complex components that are missing in tobacco leaf cells. Under the enzymatic conditions used in this study, clade A and B from the GT14 family did not demonstrate any GlcAT activity using the Gal-containing acceptors. It is possible that enzymes from these two clades of GT14 are involved in other biochemical functions that may require additional more complex acceptors to be tested. Alternatively, expressing these GTs without the C-terminal YFP tag may make these enzymes more active due to enhanced active site accessibility; it would, however, negate the ability to rapidly purify (and localise) these heterologously expressed proteins. Another possible strategy is to use a combination of GTs, e.g., co-expression of a GT31 GalT together with either a single or multiple GT14 members, as a means to reveal additional enzyme activities.

An AGP-Ca^2+^ model has been proposed whereby the negatively charged GlcA residues on AGPs are potential intramolecular Ca^2+^ binding sites on the plant cell surface that regulate cell wall integrity and are the source for intracellular Ca^2+^ signals required for the processes of plant growth and development [88]. Ca^2+^ has since been shown to bind to GlcA residues within the side chains of type II AG and that AG directly binds and releases Ca^2+^ in a pH-dependent manner [89]. Loss of function of the *GlcAT* genes *AtGlcAT14A*, *AtGlcAT14B*, and *AtGlcAT14C* led to significant reductions in Ca^2+^ binding on AGPs, which provided the first direct experimental evidence for the AGP-Ca^2+^ model [90]. Recently, Lopez-Hernandez et al. (2020) [60] analyzed higher-order mutant plants of four *Arabidopsis* GT14 family members—AtGlcAT14A, AtGlcAT14B, AtGlcAT14D, and AtGlcAT14E—to explore the impact on glucuronidation. The authors showed that in mutant plants, the GlcA decoration on AGs was similarly reduced and they bound less Ca^2+^ impacting their growth and development. Their findings suggest that the interaction between AG and Ca^2+^ is essential for several developmental processes in *Arabidopsis* and that GT14 family members play vital roles in intracellular Ca^2+^ signaling [60]. Our studies provide further key biochemical evidence for a role of a clade of GT14 family in glucuronidation of AGPs. 

In conclusion, we have provided an update of the *Arabidopsis* GT clone collection and leveraged this resource and a plant expression system for the biochemical characterization of novel GTs using the GT14 family as the exemplar. This analysis pipeline can accelerate the functional analysis of classified and non-classified GTs and help scientists to unravel the complex glycosylation processes that occur in plants.

## 4. Materials and Methods

### 4.1. Materials

The activated nucleotide sugar donors: UDP-glucuronic acid (UDP-GlcA), GDP-fucose (GDP-Fuc), UDP-glucose (UDP-Glc), UDP-galactose (UDP-Gal) were purchased from Sigma Aldrich (www.sigmaaldrich.com). UDP-Galacturonic acid (UDP-GalA), UDP-xylose (UDP-Xyl), and UDP-arabinopyranose (UDP-Ara*p*) were provided by CarboSource (Complex Carbohydrate Research Center (CCRC), Athens, GA, USA). UDP-Rhamnose (UDP-Rha) was enzymatically synthesized according to Rautengarten et al. (2014) [91]. The synthetic acceptor galactose-nitrobenzodiazole (Gal-NBD) was chemically synthesized according to McGill and Williams (2009) [61]. The galacto-oligosaccharides, β-1,6-galactotetraose (β-1,6-Gal_4_) and β-1,3-galactopentose (β-1,3-Gal_5_), were chemically synthesized according to Andersen et al. (2017) [92]. 

*Nicotiana benthamiana* (tobacco) was cultivated in a controlled environment growth room at 24 °C with a 16 h photoperiod. The leaves of 4–6 weeks old tobacco seedlings were used for the transient expression of GTs.

### 4.2. Amino Acid Sequence Alignment and Phylogenetic Analysis

Multiple alignments for the full-length amino acid sequences of GTs in the published *Arabidopsis* genome Araport11 [78] were performed using MAFFT (V7) [67,93] with default settings. Maximum likelihood (ML) phylogenetic trees were constructed using IQ-TREE [94] on XSEDE (V1.6.10) [95] with Model Finder [96] and 1000 bootstrap replicates for support values of SH-aLRT, local bootstrap, parametric aLRT, aBayes, and ultrafast bootstrap [97]. The trees were annotated using iTOL [98].

### 4.3. Analysis of Genome Collinearity and Chromosomal Localizations of GT Family Genes

The genome collinear blocks were analyzed using MCScanX [58] and TBtools (V1.0) [99], treating at least five genes as a collinear block. Each GT gene was mapped to a chromosome. Genome collinearity and chromosomal locations were drawn using TBtools and Inkscape (http://www.inkscape.org/).

### 4.4. Publicly Available Microarray Data and Sequence Architecture Analysis for the Arabidopsis GT14 Family

The public microarray data for various tissues are available at NCBI Gene Expression Omnibus (GEO) database (https://www.ncbi.nlm.nih.gov/geo/). The series accession numbers GSE34188 [59] of *Arabidopsis* from Agilent platform includes 14 different tissue samples representing three biological replicates were used for the tissue-specific expression analysis. Hybridization intensities in arrays were normalized among different arrays by quantile normalization followed by denary logarithm transformation. The data of probe set IDs corresponding to *Arabidopsis* GT14 family genes were extracted for further analyses. The GT14 gene structures were obtained using the Bath Web CD-Search Tool [100] on NCBI and the protein motifs (novel and ungapped) were found using MEME [58]. The motifs refer to the approximate sequence pattern that occurs in a group of homolog genes [100]. The gene expression and structure profiles were annotated using TBtools [99], iTOL [98], and Inkscape (http://www.inkscape.org/).

### 4.5. Arabidopsis GT Cloning

Additional GT coding DNA sequences (CDS) were cloned from *Arabidopsis thaliana* (At, Col-0) into pDONR223 according to Lao et al. (2014) [8]. An LR reaction was used to subclone each CDS into the Gateway-compatible pEarleyGate 101 binary vector containing the cauliflower mosaic virus 35S promoter and upstream enhancer and C-terminal YFP and HA tags [101]. All recombinant vectors were sequenced to verify correct assembly.

### 4.6. Heterologous Expression of GT14 Family Proteins in Tobacco

The eleven pEarleyGate 101 binary vector constructs containing each of the AtGT14 cDNAs were individually transformed into *Agrobacterium tumefaciens* strain AGL1 via electroporation. Single colonies were then cultured for transient plant expression. The abaxial surface of *N. benthamiana* leaves was coinfiltrated with a mix of *Agrobacterium* cells carrying either the GT14 construct or the P19 gene from the tomato bushy stunt virus to suppress gene silencing. The final OD600 of each strain was adjusted to 0.8 in resuspension buffer (0.01 M MgCl_2_ and 0.8 mM acetosyringone in 50 mM MES buffer pH 5.3) prior to the infiltration. After 3 days, the infected leaf areas were excised, and the fluorescence confirmed under a confocal microscope (Zeiss LSM 880, Jena, Germany). Microsomal membranes (MMs) were extracted as described by Geshi et al. (2002) [102]. In brief, about 5 g of leaves was ground using a mortar and pestle in 10 mL of chilled extraction buffer (0.1 M HEPES/KOH pH 6.8, 0.4 M sucrose, 5 mM MgCl_2_, 5 mM MnCl_2_, 1 mM phenylmethylsulfonyl fluoride; Roche EDTA-free complete protease inhibitor) and quartz sand. The homogenate was filtered through two layers of Miracloth and the filtrate was centrifuged at 3000× *g* for 20 min at 4 °C. The supernatant was then centrifuged at 30,000× *g* for 30 min at 4 °C. The MM pellets were solubilized in extraction buffer to a final concentration of 5 mg/mL and used in biochemical assays. Protein concentrations were determined with a Bradford assay using Protein Assay Dye Reagent (Bio-Rad, Hercules, CA, USA).

### 4.7. Protein Analysis

For western blot analysis, 5 µL of microsomal proteins (around 25 µg) were denatured by mixing with 15 µL 2× Laemmli sample buffer (Bio-Rad) containing 50% (*v*/*v*) β-mercaptoethanol and incubating at 70 °C for 20 min. The protein samples were then subjected to SDS-PAGE (Bio-Rad) and electroblotted onto a PVDF immobilon membrane (Merck Millipore, Billerica, MA, USA) using a Trans-Blot Turbo Transfer System (Bio-Rad). Membranes were blocked for 15 min at room temperature using TBST (Tris-buffered saline with 0.1% (*v*/*v*) Tween 20) containing 0.5% (*w*/*v*) skim milk powder filtered through a 0.22 µm filter and incubated with an anti-GFP primary antibody (MBL, Nagoya, Japan) at a 1:1000 dilution followed by a horseradish peroxidase (HRP)-conjugated secondary goat anti-rabbit IgG antibody (Proteintech, Rosemont, IL, USA) at a 1:1000 dilution. Blocking and antibody binding were performed using the SNAP i.d.^®^ 2.0 Protein Detection System. Afterwards, WesternBright ECL HRP substrate (Advansta, San Jose, CA, USA) was added to the membranes for 5 min in the dark. The HRP signal was detected by chemiluminescence using a ChemiDoc MP imaging system (Bio-Rad) and processed using the Image Lab software (Bio-Rad). *N. benthamiana* leaves infiltrated with a GFP construct were used as negative control.

### 4.8. Anthranilic Acid Labeling of Galacto-Oligosaccharides

The synthetic acceptor β-1,6-Gal_4_ and β-1,3-Gal_5_ were labeled with anthranilic acid (AA) at their reducing ends according to the method described by Alwael et al. [103]. Excess derivatization reagent AA was removed with diethyl ether three times and the AA-labeled galacto-oligosaccharides (β-1,6-Gal_4_-AA and β-1,3-Gal_5_-AA) in the lower layer separately purified using a Sep-Pak C18 cartridge (Waters, Milford, MA, USA).

### 4.9. Enzyme Assays

The *N. benthamiana* MMs were pretreated with 1% (*v*/*v*) Triton X-100 on ice prior to use them in biochemical activity assays. The reaction was performed in a total volume of 50 µL containing 5 mg/mL MMs, containing either 5 μM β-Gal-NBD or AA-labeled acceptor (β-1,6-Gal_4_-AA or β-1,3-Gal_5_-AA), 0.4 mM nucleotide sugar as a donor, and the reaction incubated at 25 °C for 2 h under constant shaking. Reactions were terminated by adding 1 μL glacial acetic acid and 1 μL 0.5 M EDTA. The reaction mixture was then centrifuged for 1 min at 13,040× *g* and the supernatant filtered using a 0.22 μm Ultrafree-MC filter (Merck Millipore, Billerica, MA, USA). The filtrate was separated on a reversed-phase (RP)-HPLC (Agilent HC-C18 Analytical 4.6 × 250 mm, 5 μm) with a fluorescence detector, using a gradient of acetonitrile (ACN)/0.1% trifluoroacetic acid (TFA): 30% ACN, 15 min; 80% ACN, 5 min; 100% ACN, 5 min; 30% ACN, 5 min for re-equilibrating at a constant flow rate (0.5 mL/min) for NBD-labeled samples (λex = 470 nm, λem = 540 nm), and a different gradient of ACN/0.1% TFA: 5–10% ACN, 10 min; 10–45% ACN, 5 min; 45% ACN, 15 min; 100% ACN, 5 min; 5% ACN, 10 min for re-equilibrating at a constant flow rate (0.5 mL/min) for AA-labeled samples (λex = 320 nm, λem = 420 nm).

### 4.10. Analysis of the Enzymatic Products by Mass Spectrometry

The GlcAT assay products with β-Gal-NBD and β-1,3-Gal_5_-AA as acceptors were fractionated and collected by RP-HPLC. The products were concentrated by a rotation vacuum concentrator (Christ RVC 2–25 CDplus, Osterode, Germany) and introduced into the ultra-performance liquid chromatography-time-of-flight mass spectrometer (Synapt-G2-Si, Waters, Milford, MA, USA) equipped with electro-spray ionization (ESI) source. The samples were directly infused through 20% mobile phase A (water, 0.1% formic acid) and 80% mobile phase B (ACN, 0.1% formic acid) at 0.2 mL/min. ESI-MS/MS spectra were operated in positive ion mode with a high voltage of 4000 V for ionization, 40 V and for sampling cone and 80 V for source offset, respectively. The source temperature was set to 100 °C and the samples were dried with high nitrogen under the temperature of 400 °C and the flow rate of 900 L/h. For MS analysis, the samples were subjected to a full scan from m/z 400 to 2000 Da and from 100 Da to 1500 Da in MS^2^ test with collision energy fixed at 32 eV.

## Supplementary Materials

The following are available online at https://www.mdpi.com/1422-0067/22/3/1360/s1, Figure S1: Chromosome location of *Arabidopsis* GT family genes. Genes from different GT families are located on chromosomes, using family-specific colors defined in Figure 2. The number of genes contained within each annotated block is indicated after the decimal point. The color of the chromosome strip shows the gene density at 100 kb intervals, lower for cool colors and higher for warm colors; Figure S2: Western-blotting analysis of the 11 recombinant AtGT14 family genes expressed in *Nicotiana benthamiana*. Lane GFP, 5 mg/mL MMs extracted from *N. benthamiana* expressing empty pEarley101 vector with GFP tag (27 kDa, 25 µg total protein). Lanes 240–250, 25 µg microsomal samples of the 11 recombinant AtGT14 family genes (240: AT1G03520, 241: AT1G53100, 242: AT1G71070, 243: AT2G37585, 244: AT3G03690, 245: AT3G15350, 246: AT3G24040, 247: AT4G03340, 248: AT4G27480, 249: AT5G15050, 250: AT5G39990); Table S1: Selected examples of *Arabidopsis* cell wall biosynthetic GTs whose catalytic specificities have been analyzed following expression in heterologous systems; Table S2: The collection of *Arabidopsis* glycosyltransferases according to CAZy (http://www.cazy.org/) and cross-referenced with the *Arabidopsis* sequences from TAIR10 (http://www.Arabidopsis.org/), Table S3: GT family genes belonging to collinearity blocks. The gene pairs are divided into two groups colored yellow and blue, and they correspond to each other in the co-linearity block. The rightmost column shows the RGB colors of their connecting lines in Figure 2 and Figure 3. All *A. thaliana* co-linearity protein pairs are displayed in second worksheet in this table.
