# Supplementary material for: A Pipeline towards the Biochemical Characterization of the Arabidopsis GT14 Family
Source: Int J Mol Sci. 2021 Jan 29;22(3):1360. doi: 10.3390/ijms22031360 (PMC7866395; doi:10.3390/ijms22031360)
Supplement: Supplementary file 1 [file ijms-22-01360-s001.zip › ijms-1068100 sm revised/Supplementary materials.pdf]

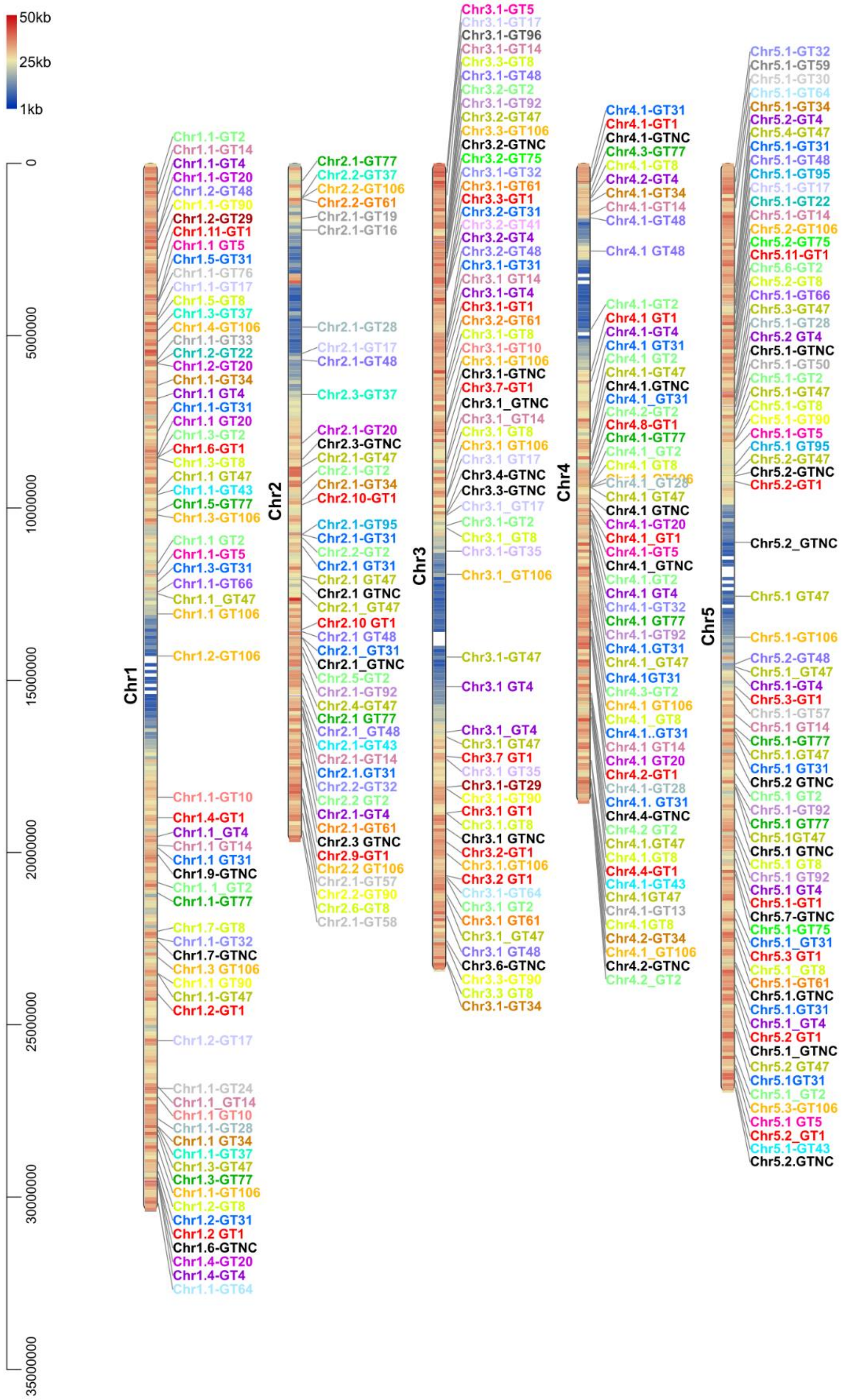

Figure S1. Chromosome location of *Arabidopsis* GT family genes. Genes from different GT families are located on chromosomes, using family-specific colors defined in Figure 2. The number of genes contained within each annotated block is indicated after the decimal point. The color of the chromosome strip shows the gene density at 100 kb intervals, lower for cool colors and higher for warm colors.

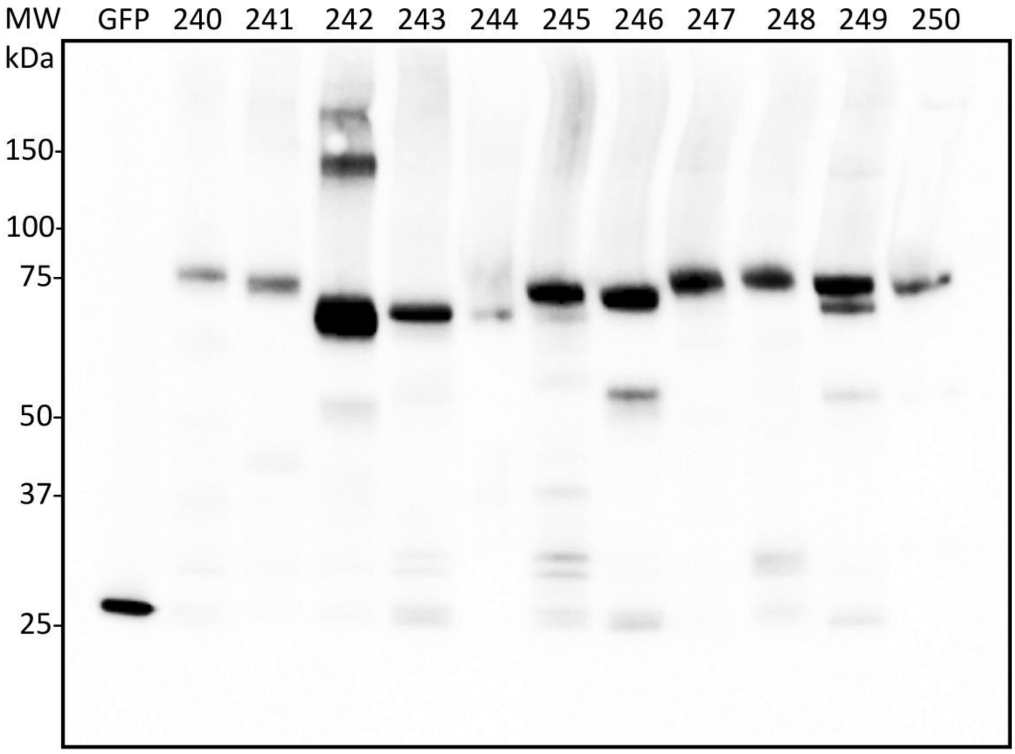

Figure S2. Western-blotting analysis of the 11 recombinant AtGT14 family genes expressed in *Nicotiana benthamiana*. Lane GFP, 5 mg/mL MMs extracted from *N. benthamiana* expressing empty pEarley101 vector with GFP tag (27 kDa, 25 µg total protein). Lanes 240-250, 25 µg microsomal samples of the eleven recombinant AtGT14 family genes (240: AT1G03520, 241: AT1G53100, 242: AT1G71070, 243: AT2G37585, 244: AT3G03690, 245: AT3G15350, 246: AT3G24040, 247: AT4G03340, 248: AT4G27480, 249: AT5G15050, 250: AT5G39990).

Table S1. Selected examples of *Arabidopsis* cell wall biosynthetic GTs whose catalytic specificities have been analyzed by following expression in heterologous systems.

GT family	Gene	Biochemical function	Expression System	Reference
2	CESA	cellulose synthase	*Pichia pastoris*	Purushotham et al. (2016). Proc. Natl. Acad. Sci. U.S.A. [1]
2	CSLA	β-1,4-mannan synthase	*Pichia pastoris*	Voiniciuc et al. (2018). Proc. Natl. Acad. Sci. U.S.A. [2]
2	CSLC	xyloglucan backbone β-1,4-glucan synthase	*Pichia pastoris*	Cocuron et al. (2007). Proc. Natl. Acad. Sci. U.S.A. [3]
2	CSLD	(gluco)mannan synthase	*Nicotiana benthamiana*	Yin et al. (2011). Mol. Plant [4]
2	CSLD3	β-1,4-glucan synthase	*Pichia pastoris*	Yang et al. (2020). Plant Cell [5]
8	GAUT	homogalacturonan α-1,4-galacturonosyltransferase	Human embryonic kidney cell line HEK293	Sterling et al. (2006). Proc. Natl. Acad. Sci. U.S.A. [6]
8	GUX1,2 and 4	xylan α-1,2-glucuronosyltransferase	*Nicotiana benthamiana*	Rennie et al.

				(2012). Plant Physiol. [7]
14	GLCAT14	AGP β-1,6-/β-1,3-galactan β-1,6-glucuronosyltransferase	*Pichia pastoris*	Knoch et al. (2013). Plant J. [8]
31	GALT	AGP β-1,3-galactosyltransferase	*Nicotiana benthamiana*	Basu et al. (2015). BMC Plant Biol. [9]
34	XXT	xyloglucan α-1,6-xylosyltransferase	*Escherichia coli*	Urbanowicz et al. (2012). Proc. Natl. Acad. Sci. U.S.A. [10] Faik et al. (2002). Proc. Natl. Acad. Sci. U.S.A. [11]
34	GMGT	galactomannan α-1,6-galactosyltransferase	*Nicotiana benthamiana*	Voiniciuc et al. (2015). Plant Physiol. [12]
37	FUT1	xyloglucan α-1,2-fucosyltransferase	Mammalian COS cells	Perrin et al. (1999). Science [13]
37	FUT4/6	AGP α-1,2-fucosyltransferase	Tobacco BY2 cells	Wu et al. (2010). J. Biol. Chem. [14]
43	IRX9/14	xylan β-1,4-xylosyltransferase	*Nicotiana benthamiana*	Zeng et al. (2016). Plant Physiol. [15]

47	IRX10	xylan β-1,4-xylosyltransferase	*Pichia pastoris*	Jensen et al. (2014). Plant J. [16]
47	MUR3	xyloglucan β-1,2-galactosyltransferase	*Pichia pastoris*	Madson et al. (2003). Plant Cell [17]
47	ARAD	arabinan α-1,5-arabinosyltransferase	*Nicotiana benthamiana*	Lampugnani et al. (2016). Plant Physiol. [18]
48	GSL	callose synthase	*Pichia pastoris*	Lars et al. (2002). Plant Mol. Biol. [19]
77	RGXT	rhamnogalacturonan-II α-1,3-xylosyltransferase	*Insect cells*	Egelund et al. (2006). Plant Cell [20]
92	GALS1,2 and 3	rhamnogalacturonan-I β-1,4-galactan synthases	*Nicotiana benthamiana*	Ebert et al. (2018). Plant Cell Physiol. [21]
9	HPAT	hydroxyproline β-arabinofuranosyltransferase	*Pichia pastoris*	Suzuki et al. (2017). Plant Physiol. [22]
106	RRT	rhamnogalacturonan-I rhamnosyltransferase	*Nicotiana benthamiana*	Takenaka et al. (2018). Nat. Plants [23]

References

1. Purushotham, P.; Cho, S.H.; Díaz-Moreno, S.M.; Kumar, M.; Nixon, B.T.; Bulone, V.; Zimmer, J. A single heterologously expressed plant cellulose synthase isoform is sufficient for cellulose microfibril formation in vitro. *Proc. Natl. Acad. Sci. U. S. A.* **2016**, *113*, 11360, doi:10.1073/pnas.1606210113.
2. Voiniciuc, C.; Dama, M.; Gawenda, N.; Stritt, F.; Pauly, M. Mechanistic insights from plant heteromannan synthesis in yeast. *Proc. Natl. Acad. Sci. U. S. A.* **2019**, *116*, 522-527, doi:10.1073/pnas.1814003116.
3. Cocuron, J.C.; Lerouxel, O.; Drakakaki, G.; Alonso, A.P.; Liepman, A.H.; Keegstra, K.; Raikhel, N.; Wilkerson, C.G. A gene from the cellulose synthase-like C family encodes a β-1,4 glucan synthase. *Proc. Natl. Acad. Sci. U. S. A.* **2007**, *104*, 8550-8555, doi:10.1073/pnas.0703133104.
4. Yin, L.; Verhertbruggen, Y.; Oikawa, A.; Manisseri, C.; Knierim, B.; Prak, L.; Jensen, J.K.; Knox, J.P.; Auer, M.; Willats, W.G., et al. The cooperative activities of CSLD2, CSLD3, and CSLD5 are required for normal *Arabidopsis* development. *Mol. Plant.* **2011**, *4*, 1024-1037, doi:10.1093/mp/ssr026.
5. Yang, J.; Bak, G.; Burgin, T.; Barnes, W.J.; Mayes, H.B.; Pena, M.J.; Urbanowicz, B.R.; Nielsen, E. Biochemical and genetic analysis identify CSLD3 as a beta-1,4-glucan synthase that functions during plant cell wall synthesis. *Plant Cell* **2020**, *32*, 1749-1767, doi:10.1105/tpc.19.00637.
6. Sterling, J.D.; Atmodjo, M.A.; Inwood, S.E.; Kumar Kolli, V.S.; Quigley, H.F.; Hahn, M.G.; Mohnen, D. Functional identification of an *Arabidopsis* pectin biosynthetic homogalacturonan galacturonosyltransferase. *Proc. Natl. Acad. Sci. U. S. A.* **2006**, *103*, 5236-5241, doi:10.1073/pnas.0600120103.
7. Rennie, E.A.; Hansen, S.F.; Baidoo, E.E.; Hadi, M.Z.; Keasling, J.D.; Scheller, H.V. Three members of the Arabidopsis glycosyltransferase family 8 are xylan glucuronosyltransferases. *Plant Physiol.* **2012**, *159*, 1408-1417, doi:10.1104/pp.112.200964.
8. Knoch, E.; Dilokpimol, A.; Tryfona, T.; Poulsen, C.P.; Xiong, G.; Harholt, J.; Petersen, B.L.; Ulvskov, P.; Hadi, M.Z.; Kotake, T., et al. A β-glucuronosyltransferase from *Arabidopsis thaliana* involved in biosynthesis of type II arabinogalactan has a role in cell elongation during seedling growth. *Plant J.* **2013**, *76*, 1016-1029, doi:10.1111/tpj.12353.
9. Basu, D.; Tian, L.; Wang, W.; Bobbs, S.; Herock, H.; Travers, A.; Showalter, A.M. A small multigene hydroxyproline-*O*-galactosyltransferase family functions in arabinogalactan-protein glycosylation, growth and development in *Arabidopsis*. *BMC Plant Biol.* **2015**, *15*, 295, doi:10.1186/s12870-015-0670-7.
10. Urbanowicz, B.R.; Pena, M.J.; Ratnaparkhe, S.; Avci, U.; Backe, J.; Steet, H.F.; Foston, M.; Li, H.; O'Neill, M.A.; Ragauskas, A.J., et al. 4-*O*-methylation of glucuronic acid in Arabidopsis glucuronoxylan is catalyzed by a domain of unknown function family 579 protein. *Proc. Natl. Acad. Sci. U. S. A.* **2012**, *109*, 14253-14258, doi:10.1073/pnas.1208097109.
11. Faik, A.; Price, N.J.; Raikhel, N.V.; Keegstra, K. An *Arabidopsis* gene encoding an *α*-xylosyltransferase involved in xyloglucan biosynthesis. *Proc. Natl. Acad. Sci. U. S. A.*

2002, *99*, 7797-7802, doi:10.1073/pnas.102644799.
12. Voiniciuc, C.; Schmidt, M.H.; Berger, A.; Yang, B.; Ebert, B.; Scheller, H.V.; North, H.M.; Usadel, B.; Gunl, M. MUCILAGE-RELATED10 produces galactoglucomannan that maintains pectin and cellulose architecture in *Arabidopsis* seed mucilage. *Plant Physiol.* **2015**, *169*, 403-420, doi:10.1104/pp.15.00851.
13. Perrin, R.M.; DeRocher, A.E.; Bar-Peled, M.; Zeng, W.; Norambuena, L.; Orellana, A.; Raikhel, N.V.; Keegstra, K. Xyloglucan fucosyltransferase, an enzyme involved in plant cell wall biosynthesis. *Science* **1999**, *284*, 1976-1979, doi:10.1126/science.284.5422.1976.
14. Wu, Y.; Williams, M.; Bernard, S.; Driouich, A.; Showalter, A.M.; Faik, A. Functional identification of two nonredundant *Arabidopsis* α(1,2)fucosyltransferases specific to arabinogalactan proteins. *J. Biol. Chem.* **2010**, *285*, 13638-13645, doi:10.1074/jbc.M110.102715.
15. Zeng, W.; Lampugnani, E.R.; Picard, K.L.; Song, L.; Wu, A.M.; Farion, I.M.; Zhao, J.; Ford, K.; Doblin, M.S.; Bacic, A. Asparagus IRX9, IRX10, and IRX14A are components of an active xylan backbone synthase complex that forms in the Golgi apparatus. *Plant Physiol.* **2016**, *171*, 93-109, doi:10.1104/pp.15.01919.
16. Jensen, J.K.; Johnson, N.R.; Wilkerson, C.G. *Arabidopsis thaliana* IRX10 and two related proteins from psyllium and *Physcomitrella patens* are xylan xylosyltransferases. *Plant J.* **2014**, *80*, 207-215, doi:10.1111/tpj.12641.
17. Madson, M.; Dunand, C.; Li, X.; Verma, R.; Vanzin, G.F.; Caplan, J.; Shoue, D.A.; Carpita, N.C.; Reiter, W.D. The MUR3 gene of *Arabidopsis* encodes a xyloglucan galactosyltransferase that is evolutionarily related to animal exostosins. *Plant Cell* **2003**, *15*, 1662-1670.
18. Lampugnani, E.R.; Ho, Y.Y.; Moller, I.E.; Koh, P.L.; Golz, J.F.; Bacic, A.; Newbigin, E. A glycosyltransferase from *Nicotiana alata* pollen mediates synthesis of a linear (1,5)-alpha-L-arabinan when expressed in *Arabidopsis*. *Plant Physiol.* **2016**, *170*, 1962-1974, doi:10.1104/pp.15.02005.
19. Lars, Ø.; Petersen, M.; Mattsson, O.; Mundy, J. An *Arabidopsis* callose synthase. *Plant Mol. Biol.* **2002**, *49*, 559-566.
20. Egelund, J.; Petersen, B.L.; Motawia, M.S.; Damager, I.; Faik, A.; Olsen, C.E.; Ishii, T.; Clausen, H.; Ulvskov, P.; Geshi, N. *Arabidopsis thaliana* RGXT1 and RGXT2 encode Golgi-localized (1,3)-α-D-xylosyltransferases involved in the synthesis of pectic rhamnogalacturonan-II. *Plant Cell* **2006**, *18*, 2593-2607, doi:10.1105/tpc.105.036566.
21. Ebert, B.; Birdseye, D.; Liwanag, A.J.M.; Laursen, T.; Rennie, E.A.; Guo, X.; Catena, M.; Rautengarten, C.; Stonebloom, S.H.; Gluza, P., et al. The three members of the *Arabidopsis* glycosyltransferase family 92 are functional β-1,4-galactan synthases. *Plant Cell Physiol.* **2018**, *59*, 2624-2636, doi:10.1093/pcp/pcy180.
22. Suzuki, T.; Narciso, J.O.; Zeng, W.; van de Meene, A.; Yasutomi, M.; Takemura, S.; Lampugnani, E.R.; Doblin, M.S.; Bacic, A.; Ishiguro, S. KNS4/UPEX1: a type II arabinogalactan beta-(1,3)-galactosyltransferase required for pollen exine development. *Plant Physiol.* **2017**, *173*, 183-205, doi:10.1104/pp.16.01385.
23. Takenaka, Y.; Kato, K.; Ogawa-Ohnishi, M.; Tsuruhama, K.; Kajiura, H.; Yagyu, K.; Takeda, A.; Takeda, Y.; Kunieda, T.; Hara-Nishimura, I., et al. Pectin RG-I rhamnosyltransferases represent a novel plant-specific glycosyltransferase family. *Nat.*

Plants **2018**, *4*, 669-676, doi:10.1038/s41477-018-0217-7.
